# More bricks in the wall against SARS-CoV-2 infection: involvement of γ9δ2 T cells

**DOI:** 10.1038/s41423-020-0473-0

**Published:** 2020-05-28

**Authors:** Ger Rijkers, Trees Vervenne, Pieter van der Pol

**Affiliations:** 1 St. Elisabeth Hospital, Tilburg, The Netherlands; 2 Admiral De Ruyter Hospital, Goes, The Netherlands; 3 Science Department, University College Roosevelt, Middelburg, The Netherlands

**Keywords:** Prognostic markers, Gammadelta T cells

The SARS-CoV-2 virus which emerged in late December 2019 had reached pandemic proportions by March 2020.^1^ Host defence mechanisms against this new member of the corona virus family will include innate immunity, humoral, and cellular immune responses, of yet unknown relative importance. Conventional CD8^+^ αβTCR cytotoxic T cells and natural killer cells are mainly responsible for detection and elimination of virus infected cells, with a special role for the CD94/NK group 2 member A (NKG2A) receptor as reported by Zheng et al. in this journal.^2,3^ We want to report yet another brick in the wall against SARS-CoV-2 infection, made of a subset of γδTCR T cells.^4^

Poccia et al. previously described that in peripheral blood of health care workers who survived a SARS-CoV infection during the 2003 outbreak, a selective expansion of the Vγ9Vδ2 T-cell population was found 3 months after the onset of disease.^4^ This subset of γδ T cells also has been implicated in influenza infections.^5,6^ We have therefore analyzed the frequency and activation status of Vγ9Vδ2 T cells in hospitalized patients (*n* = 24) with PCR proven SARS-CoV-2 infection (Supplementary Table 1). We find that the percentage of Vγ9Vδ2 T cells at the moment of hospital admission (on average 10 days after onset of clinical symptoms) is significantly lower than that of matched healthy controls (Fig. 1) (healthy controls 1.82 ± 0.41 × 10^4^ Vγ9Vδ2 T cells/ml, COVID-19 patients 0.38 ± 0.40 × 10^4^/ml ; *p* < 0.05). Six patients died while being hospitalized (four of them in the ICU) and they showed T lymphocytopenia, including decreased numbers of Vγ9Vδ2 T cells (0.06 ± 0.38 × 10^4^/ml; Fig. 1). In five patients we could monitor the phenotype of Vγ9Vδ2 T cells during the 2 weeks they were admitted to the hospital. During that period, on average 26% of the Vγ9Vδ2 T-cell population shifts to a phenotype of effector (memory) cells, as compared with 8% within the total T-cell population.

**Fig. 1:**
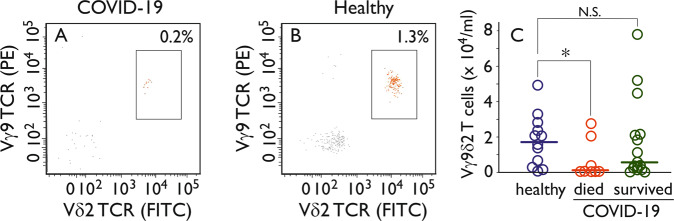
Reduced Vγ9Vδ2 T-cell numbers in SARS-CoV-2 infected patients with fatal outcome. Heparinized peripheral blood was incubated with a combination of CD45 V500, CD3 PerCP, CD4 PECy7, CD8 APC-H7, Vγ9 TCR PE, and Vδ2 TCR FITC labeled antibodies (see specifications in Supplementary Table 2) and measured by flow cytometry on a BD FACSLyric instrument (BD Biosciences, San Jose, CA, USA). Data analysis was performed with Infinicyt flow cytometry software (Cytognos, Capelle aan de IJssel, The Netherlands). Lymphocytes were gated on basis of CD45 and scatter characteristics. **a**, **b** Show the percentage of CD3^+^, CD4^−^, CD8 bright^−^ T-lymphocytes expressing Vγ9 TCR and/or Vδ2 TCR of a representative COVID-19 patient and a healthy control donor, respectively. The boxed areas represents the Vγ9Vδ2 TCR T lymphocytes. **c** The numbers of Vγ9Vδ2 TCR T lymphocytes per ml are given for healthy controls and COVID-19 patients that died or survived. Patients who died of COVID-19 had a significant lower number of Vγ9Vδ2 TCR T lymphocytes (*p < 0.05 by two-sided Student *T* test). N.S. not significant

It has been shown that Vγ9Vδ2 T cells have a so-called polycytotoxic profile.^6^ Vγ9Vδ2 cells are the dominant γδ T-cell population in adults, but in the elderly this is more variable.^6,7^ Our data could indicate that elderly with reduced numbers of Vγ9Vδ2 T cells constitute the SARS-CoV-2 vulnerable population. Alternatively, the Vγ9Vδ2 T cells in these patients have migrated to the lungs to kill SARS-CoV-2 infected cells. Long term monitoring of these patients should make this clear.

Vγ9Vδ2 T cells do not recognize antigens presented by HLA molecules but use the alternative antigen presenting molecule BTN3A.^8^ ICT01, a humanized activating anti-BTN3A antibody, is currently in Phase 1 studies for potential use in anticancer therapy.^9^ In the context of the data presented here, this antibody could offer an alternative treatment strategy for COVID-19.

The study was performed in accordance with the guidelines for sharing of patient data of observational scientific research in emergency situations as issued by the Commission on Codes of Conduct of the Foundation Federation of Dutch Medical Scientific Societies (https://www.federa.org/federa-english).

## Supplementary information

Supplementary Tables
