# Supplementary material for: More bricks in the wall against SARS-CoV-2 infection: involvement of γ9δ2 T cells
Source: Cell Mol Immunol. 2020 May 28;17(7):771–2. doi: 10.1038/s41423-020-0473-0 (PMC7331628; doi:10.1038/s41423-020-0473-0)
Supplement: Supplementary file 1 — Supplementary Tables [file 41423_2020_473_MOESM1_ESM.docx]

Supplementary Table 1. Patient characteristics

|  | Total | Died | Recovered | Significance |
| --- | --- | --- | --- | --- |
| N (%) | 24 (100) | 6 (25) | 18 (75) |  |
| Male sex | 19 (79) | 6 (100) | 13 (72) | 0.014 |
| Age in years (range) | 70 (47-88) | 75 (64-88) | 68 (47-87) |  |
| Body mass index (range) | 27 (19-42) | 28 (19-42) | 26 (21-33) |  |
|  |  |  |  |  |
| Intensive care (%) | 13 (54) | 4 (67) | 9 (50) |  |
| Days on IC (range) | 16 (1-29) | 22 (15-29) | 13 (1-23) | 0.040 |
|  |  |  |  |  |
| Leukocytes (x 10^-3^/μl) ± S.D. | 11.2 ± 6.9 | 15.2 ± 7.7 | 9.8 ± 6.3 |  |
| % CD3^+^ T lymphocytes ± S.D. | 11.9 ± 8.6 | 4.8 ± 2.9 | 14.3 ± 8.6 | < 0.001 |
| CD3^+^ T lymphocytes  (x 10^-3^/μl) ± S.D. | 1.18 ± 1.0 | 0.85 ± 0.70 | 1.30 ± 1.0 |  |
|  |  |  |  |  |

Supplementary Table 2. mAbs for flow cytometry

| Antibody | Fluorochrome | Clone | Source |
| --- | --- | --- | --- |
| Panel Vγ9δ2 T lymphocytes |  |  |  |
| CD45 | V500 | B6 | BD Biosciences |
| CD3 | PerCP | SK7 | BD Biosciences |
| CD4 | PECy7 | SK3 | BD Biosciences |
| CD8 | APC-H7 | SK1 | BD Biosciences |
| CD27 | APC | M-T271 | BD Biosciences |
| Vγ9 TCR | FITC | B3 | BD Biosciences |
| Vδ2 TCR | PE | B6 | BD Biosciences |
|  |  |  |  |
| Panel CD45RA CD45RO |  |  |  |
| CD3 | PerCP | SK7 | BD Biosciences |
| CD4 | PECy7 | SK3 | BD Biosciences |
| CD8 | APC-H7 | SK1 | BD Biosciences |
| CD27 | APC | M-T271 | BD Biosciences |
| CD45RA | FITC | L48 | BD Biosciences |
| CD45RO | PE | UCHL1 | BD Biosciences |
